# The Screening and Correlation of Trace Elements in the Blood and Urine of School-Aged Children (5–12 Years): A Pilot Biomonitoring Study

**DOI:** 10.3390/toxics13060431

**Published:** 2025-05-25

**Authors:** Arlette A. Camacho-delaCruz, Oliver Mendoza-Cano, Xóchitl Trujillo, Miguel Huerta, Mónica Ríos-Silva, Irma Elizabeth Gonzalez-Curiel, Agustin Lugo-Radillo, María Fernanda Romo-García, Herguin Benjamin Cuevas-Arellano, Ángel Gabriel Hilerio-López, Ramón Solano-Barajas, Jaime Alberto Bricio-Barrios, Juan Manuel Uribe-Ramos, J. Francisco Ventura-Ramírez, Alma Alejandra Solano-Mendoza, Fernando Sánchez-Cárdenas, Verónica Benites-Godínez, Eder Fernando Ríos-Bracamontes, Jesús Venegas-Ramírez, Efrén Murillo-Zamora

**Affiliations:** 1 Facultad de Ingeniería Civil, Universidad de Colima, Carretera Colima-Coquimatlán km 9, Coquimatlán 28400, Mexico; 2 Centro Universitario de Investigaciones Biomédicas, Universidad de Colima, Av. 25 de Julio 965, Colima 28045, Mexico; 3 Facultad de Medicina, Universidad de Colima, Av. Universidad 333, Colima 28040, Mexico; 4 Laboratorio de Inmunotoxicología, Unidad Académica de Ciencias Químicas, Universidad Autónoma de Zacatecas, Campus UAZ Siglo XXI, Carretera Zacatecas-Guadalajara km 6, Zacatecas 98160, Mexico; 5 SECIHTI—Facultad de Medicina y Cirugía, Universidad Autónoma Benito Juárez de Oaxaca, Ex Hacienda Aguilera S/N, Carr. a San Felipe del Agua, Oaxaca 68020, Mexico; 6 Facultad de Ciencias, Universidad de Colima, Bernal Díaz del Castillo 340, Colima 28045, Mexico; 7 Facultad de Enfermería, Universidad de Colima, Av. Universidad 333, Colima 28040, Mexico; 8 Secretaría Técnica de la Rectoría, Universidad de Colima, Av. Universidad 333, Colima 28040, Mexico; 9 Departamento de Medicina Interna, Hospital Civil de Guadalajara “Juan I. Menchaca”, Universidad de Guadalajara, Salvador Quevedo y Zubieta 750, Guadalajara 44340, Mexico; 10 Coordinación General de Investigación, Universidad de Colima, Av. Universidad 333, Colima 28040, Mexico; 11 Coordinación de Educación en Salud, Jefatura de Servicios de Prestaciones Médicas, Instituto Mexicano del Seguro Social, Calzada del Ejército Nacional No. 14, Tepic 63169, Mexico; 12 Unidad Académica de Medicina, Universidad Autónoma de Nayarit, Ciudad de La Cultura Amado Nervo s/n, Tepic 63000, Mexico; 13 Departamento de Medicina Interna, Hospital General de Zona No. 1, Instituto Mexicano del Seguro Social, Av. Lapislázuli 250, Villa de Álvarez 28984, Mexico; 14 Coordinación Auxiliar de Investigación en Salud, Jefatura de Servicios de Prestaciones Médicas, Instituto Mexicano del Seguro Social, Doroteo López 442, Colima 28030, Mexico; 15 Unidad de Investigación en Epidemiología Clínica, Instituto Mexicano del Seguro Social, Av. Lapislázuli 250, Villa de Álvarez 28984, Mexico

**Keywords:** environmental exposure, trace elements, child, biological monitoring

## Abstract

Children constitute a population at risk from environmental exposure to trace elements. This study aimed to evaluate correlations between urinary and blood levels of multiple elements in school-aged children (5–12 years), assessing whether urine, a less invasive matrix, could complement or replace blood sampling. A pilot biomonitoring study was conducted, and 91 children provided urine and venous blood samples in which the levels of 17 contaminants (Al, As, Ba, Cs, Co, Cu, I, Pb, Li, Mn, Mo, Ni, Se, Sr, Te, Ti, and Zn) were assessed. Spearman correlation coefficients (rho) and 95% confidence intervals (CI) were computed. Urinary and blood levels of arsenic (rho = 0.23, 95% CI 0.01–0.44), lead (rho = 0.43, 95% CI 0.24–0.61), and strontium (rho = 0.22, 95% CI 0.03–0.40) showed significant correlations. These findings suggest that urine sampling could serve as a practical alternative to blood collection for monitoring specific trace elements like lead in pediatric populations, particularly in large-scale studies where participant compliance is critical. However, modest correlations for other elements highlight the need for element-specific validation before adopting urine as a universal biomonitoring matrix. Future research should explore the pharmacokinetic and exposure-related factors driving these relationships to optimize non-invasive surveillance strategies for children’s environmental health.

## 1. Introduction

Children are a population vulnerable to the adverse health effects of environmental exposure to trace elements, due to their physiological characteristics, developmental stages, and behaviors that increase exposure risks [1]. The exposure to these contaminants has been associated with neurodevelopmental disorders, cognitive deficits, and various systemic effects [2,3,4,5], among others. Understanding the extent of exposure and its potential health impacts is crucial for implementing effective preventive measures [6].

Biomonitoring studies often use blood or urine to quantify the level of exposure to trace elements [7]. However, few studies have systematically evaluated the correlation between the blood and urinary levels of these elements in pediatric populations [8,9,10]. Establishing such correlations is critical: if metal concentrations in urine strongly reflect those in blood, the gold-standard matrix for many elements, this would justify prioritizing non-invasive urine sampling for large-scale pediatric biomonitoring, thereby reducing participant burden and improving compliance in vulnerable age groups. In addition, a significant blood–urine correlation may suggest consistent exposure kinetics, which could arise from persistent sources (e.g., dietary or environmental contamination). However, this relationship must be interpreted alongside metal-specific pharmacokinetics and temporal exposure patterns [11].

Although one of the smallest states in Mexico, Colima, located along the Pacific coast in the western region of the country, features a range of economic activities. These include sugarcane production in Quesería, large-scale agriculture in Tecomán and Armería, cement production in Caleras, mining in Minatitlán and Paticajo, major port-related activities in Manzanillo, and thermal power generation in Campos. These industries have the potential to produce toxic substances, including trace elements [12].

This study aimed to evaluate the correlation between the measured urinary and blood levels of 17 trace elements (aluminum, arsenic, barium, cesium, cobalt, copper, iodine, lead, lithium, manganese, molybdenum, nickel, selenium, strontium, tellurium, titanium, and zinc) in school-aged (5–12 years) children residing in areas with potential environmental exposure to these contaminants. This research is part of broader endeavors to comprehend and mitigate the health risks associated with environmental exposures in children, ultimately aiming to inform policies and interventions that safeguard children’s health and well-being.

## 2. Materials and Methods

### 2.1. Study Design

From September to October 2023, a pilot biomonitoring study was conducted across nine urban and rural communities in the state of Colima, Mexico: the urban area of Colima—Villa de Álvarez (the state capital), Caleras, Tecomán, Armería, Campos, Manzanillo, Paticajo, Minatitlán, and Quesería.

These localities were selected based on environmental risk stratification. They were classified as high-risk areas due to their proximity to industrial corridors (e.g., mining operations, thermal power plants, and agricultural chemical use zones), with exposure potentials corroborated by atmospheric dispersion models [13].

### 2.2. Study Population

Participants were selected using a non-probabilistic, multistage approach. Initially, elementary schools in high-risk areas were identified, followed by the selection of eligible children from these schools. We conducted informational workshops at each school where parents/guardians received study details. Children were enrolled if they (1) attended with a parent/guardian, and (2) met all eligibility criteria.

### 2.3. Eligibility Criteria

Study participants were children aged 5 to 12 years who had lived in participating localities for at least five years, as reported by a parent or legal guardian. Exclusions were made for children with chronic non-communicable diseases, and those who had consumed processed foods within 24 h prior to urine sample collection to avoid potential contamination. For dietary assessment, we asked the parents/guardians of potentially eligible participants: “In the past 24 h, did your child consume any of the following processed foods?” The response options were: packaged snacks (e.g., chips, cookies), sugary beverages (e.g., sodas, energy drinks), instant meals (e.g., microwaveable dinners, instant noodles), processed meats (e.g., hot dogs), frozen meals or snacks, other, and none. The 24 h exclusion of processed foods was implemented to minimize acute dietary confounding of urinary metal levels, as many processed foods (e.g., canned goods, snacks with additives) can transiently elevate urinary concentrations of certain elements (e.g., Al, Pb, Sn). This approach aligns with studies prioritizing acute exposure differentiation from background levels.

### 2.4. Laboratory Methods

Morning spot-urine samples were collected via spontaneous micturition in sterile containers (150 mL), used for quantifying specific gravity, and then stored at −4 °C until metal and metalloid determination. Blood samples (4 mL) were collected via peripheral venipuncture into sterile tubes of BD Vacutainer^®^ with K2 ethylenediaminetetraacetic acid (EDTA; lavender cap) with purple caps and stored at 4 °C until metal and metalloid quantification.

The urine samples were thawed, and aliquots of 200 μL were directly diluted 1:10 with 65% nitric acid (Merck, Darmstadt, Hesse, Germany) and ultrapure water to achieve a final acid concentration of 0.16% (*v*/*v*). The blood samples underwent the acid digestion process with microwave irradiation using Milestone Ethos One equipment (Milestone S.r.l., Sorisole, Italy) prior to element quantification. The samples were then analyzed using an ICP-MS NexION 300D (Perkin Elmer, Waltham, MA, USA). The equipment was optimized for each run according to the manufacturer’s instructions. Quantification was performed using a validated method (LISTO-MET-PRO-TEC-016) developed by the Metal Laboratory of the Research and Service Laboratory in Toxicology at the Center for Research and Advanced Studies of the National Polytechnic Institute (CINVESTAV) of Mexico. Measurements were conducted in duplicate, and the analysis included a calibration curve consisting of a blank and at least six concentrations (0.5, 1, 5, 10, 25, 50, and 100 ng/mL) prepared from multi-element calibration standards 2, 3, 4, and 5 (P/N N9300232, N9300233, N9300234, and N9300235; Perkin Elmer, Waltham, MA, USA). The full set of the employed ICP-MS parameters is presented as Supplementary Materials.

For quality control, the precision and accuracy of the determinations were assessed, ensuring that the analytical coefficient of variation did not exceed 10% in sample duplicates. Accuracy was evaluated using reference materials for urine (QM-U-Q2104, 2105, 2106, 2113, 2114, and 2115) and for blood (QM-B-Q2119, 2120, 2121, 2201, 2202, 2203, 2401, 2402, and 2403), provided by the Institut National de Santé Publique du Québec (Québec, QC, Canada). These materials were analyzed alongside the study samples, achieving an accuracy range of 80–120%.

Additionally, the measured concentrations of trace elements in urine were adjusted for specific gravity by dividing the measured concentrations by the specific gravity levels of the urine samples.

For children with undetectable levels (≤limit of detection, LOD) of any analyzed trace elements in either body fluid, exposure levels were imputed as LOD/√2. The imputation method provides a conservative estimate that minimizes the potential impact of undetected values on the overall analysis, thereby allowing for a more robust interpretation of the data [14]. The following counts of imputed values were based on the LOD in each body fluid: aluminum (LOD ≤ 0.045 ng/mL; 4 in urine and 8 in blood), arsenic (LOD ≤ 0.041 ng/mL; 6 in urine), barium (LOD ≤ 0.060 ng/mL; 44 in blood), cobalt (LOD ≤ 0.053 ng/mL; 4 in urine and 30 in blood), copper (LOD ≤ 0.112 ng/mL; 4 in urine), lead (LOD ≤ 0.065 ng/mL; 21 in blood), manganese (LOD ≤ 0.065 ng/mL; 42 in urine), molybdenum (LOD ≤ 0.071 ng/mL; 2 in blood), nickel (LOD ≤ 0.043 ng/mL; 2 in urine and 32 in blood), strontium (LOD ≤ 0.529 ng/mL; 1 in urine and 45 in blood), and titanium (LOD ≤ 0.174 ng/mL; 2 in urine and 9 in blood).

### 2.5. Statistical Analysis

Summary statistics were computed for the variables of interest. The distributions of element concentrations were assessed for skewness using Shapiro–Wilk tests. To assess the relationship between urinary and blood levels of the 17 trace elements included in the study, Spearman’s correlation coefficients (rho) and their respective 95% confidence intervals were calculated. Spearman’s method was selected due to its non-parametric nature, which accommodates non-normal distributions and potential outliers common in environmental biomarker data. All analyses were conducted by using Stata 17.0 MP (StataCorp; College Station, TX, USA).

### 2.6. Ethical Considerations

Informed consent from a parent or guardian and child assent were required. The study protocol was reviewed and approved by the Committee of Ethics in Health Research of the Ministry of Health in the state where the study was conducted (approval CBCANCL2306023-PRONAII-17). All procedures adhered to the relevant ethical guidelines and regulations.

## 3. Results

Data from 91 children were analyzed. Among them, 50.6% (n = 46) were female. The mean age of the children was 8.2 ± 1.5 years. No significant differences in age were observed between females and males (*p* = 0.582).

Table 1 summarizes the median levels of both the blood and urine levels of the 17 evaluated trace elements. Arsenic (rho = 0.23, 95% CI 0.01–0.44; *p* = 0.039), lead (rho = 0.43, 95% CI 0.24–0.61; *p* < 0.001), and strontium (rho = 0.22, 95% CI 0.03–0.40, *p* = 0.023) showed significant positive correlations between the levels in both biological fluids. We did not observe correlations for the other trace elements (Table 1).

**Figure 1:**
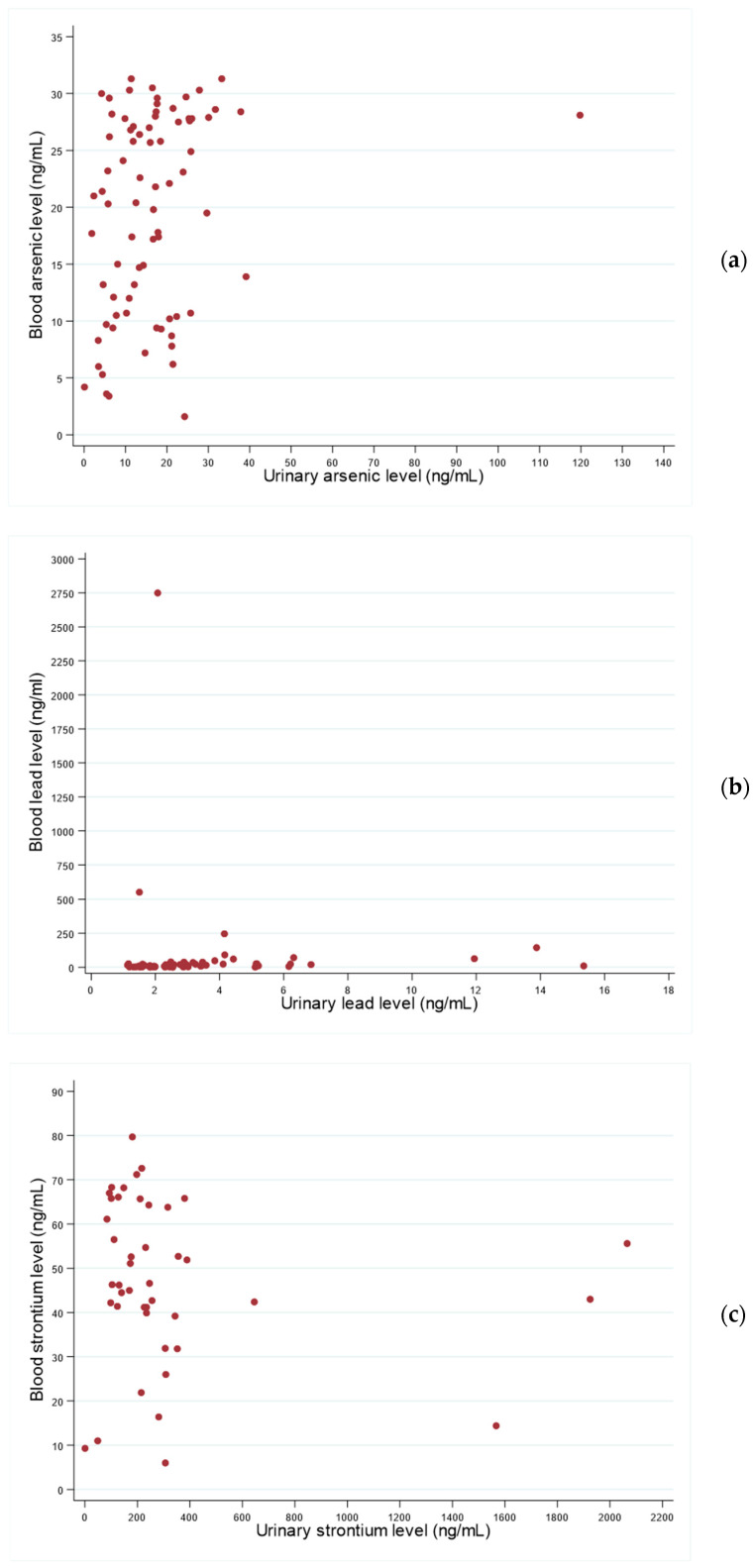
(**a**–**c**) Urinary and blood levels of arsenic (**a**), lead (**b**), and strontium (**c**) in school-aged (5–12 years) children, Mexico, 2023. Note: (1) Spearman’s correlation coefficients (rho), and 95% confidence intervals (CI) were as follows: arsenic, rho = 0.29 (95% CI 0.01–0.44); lead, rho = 0.43 (95% CI 0.24–0.41); and strontium rho = 0.22, (95% CI 0.03–0.40); (2) In (**a**), if the participant with unusually high urinary and blood levels of arsenic (119.8 and 28.1 ng/mL, respectively) is excluded, the correlation coefficient remains similar (rho = 0.27, 95% CI 0.04–0.50); (3) In (**b**), if the participant with unusually high blood levels of lead (2743.3 ng/mL) is excluded, the correlation coefficient remains similar (rho = 0.43, 95% CI 0.22–0.65); (4) In (**c**), if the participants with unusually high blood levels of strontium (>1556.0 ng/mL) are excluded, the correlation coefficient remains similar (rho = 0.21, 95% CI 0.01–0.41).

The measured urinary and blood levels of arsenic, lead, and strontium are presented in Figure 1a–c. One female child exhibited unusually high levels of arsenic in both urine (119.8 ng/mL) and blood (28.1 ng/mL). To assess the influence of this potential outlier, we performed the correlation analysis again after excluding this participant. The correlation coefficient remained similar (rho = 0.27, 95% CI 0.04–0.50; *p* = 0.020). Additionally, we identified a male participant with an unexpectedly high blood lead level (2743.3 ng/mL). Excluding this participant from the correlation analysis also had minimal impact, with the estimate remaining significant (rho = 0.43, 95% CI 0.22–0.65; *p* < 0.001). The urinary lead level in this male participant was 2.1 ng/mL.

Finally, we identified three girls, each from different localities (Minatitlán, Campos, and Tecomán), with urinary strontium levels exceeding 1500 ng/mL (1556.3; 1566.7; and 1924.7 ng/mL, respectively). After excluding these participants from the analysis, the correlation coefficient remained significant (rho = 0.21, 95% CI 0.01–0.41; *p* = 0.042).

## 4. Discussion

Our study provides a comprehensive analysis of environmental exposure to trace elements in a sample of children residing in areas with environmental risks. The results indicate that the blood and urinary levels of three substances (arsenic, lead, and strontium) exhibit correlations; however, it is important to note that Spearman’s rho values suggest weak to very weak associations (rho < 0.5), which may even be negligible in some cases. While these correlations could be secondary to industrial and other productive activities in the children’s residential areas, the modest strength of the associations implies that other unmeasured factors may also contribute to exposure variability. Thus, caution should be exercised when interpreting these findings, and further research is needed to clarify potential exposure pathways [15].

The half-life of environmental arsenic in urine and blood in children can vary depending on several factors, including the specific arsenic compounds present and individual metabolic differences. Generally, inorganic arsenic, which is considered more toxic [16], has a shorter half-life in urine (approximately 2–4 days) compared to organic arsenic compounds [17]. In blood, arsenic clearance is rapid, with total elimination typically occurring within 2–3 days [18].

Arsenic exposure has been extensively studied, particularly regarding its neurotoxic effects on children. Published research indicates that long-term exposure to arsenic is associated with cognitive impairments, including deficits in memory and executive functioning [19]. However, the study by Vega-Millán et al. did not find a significant correlation between urinary arsenic levels and health outcomes, suggesting that the relationship may be complex [20]. Urinary arsenic concentration is the most widely accepted biomarker for assessing exposure to this element. However, data on reference concentrations of urinary arsenic for environmental exposure in children remain limited. A study published in 2018 by Limón-Pacheco et al. proposed a Biomonitoring Equivalent (BE) of 15 µg/L (15 ng/mL) for urinary arsenic in the Mexican pediatric population, indicating a concentration associated with an acceptable level of exposure [21]. In the present study, the median urinary arsenic concentration was found to be 13.4 ng/mL, which is below the 15 ng/mL threshold. Nevertheless, about 28% of study participants exhibited higher concentrations (≥20 ng/mL), and these cases have been monitored accordingly.

In children, the half-life of environmental lead in urine blood averages around 30 days [9]. However, around 90% of lead is distributed in the bone, where it has a half-life of over 20 years [22]. Elevated blood lead levels can adversely affect cognitive development [23]. Blood lead concentration is the most widely used biomarker of lead exposure, though it accounts for only about 1% of the total body lead burden. It reflects both recent external lead exposure and the endogenous redistribution of lead from bone [24].

There are no safe levels of exposure to contaminants such as lead. The World Health Organization (WHO) reports that even at blood lead concentrations below 50 ng/mL (5 µg/dL), environmentally exposed children may experience adverse effects, including reduced IQ, cognitive impairment, lower academic performance, increased incidence of behavioral problems, and a higher likelihood of attention deficit hyperactivity disorder diagnoses [24].

In the present study, the median blood lead concentration in children was 11.3 ng/mL, which is below the 50 ng/mL threshold. However, 9% of the participants had blood lead concentrations exceeding 50 ng/mL, and these cases have also been closely monitored.

Strontium, while less frequently discussed in the context of toxicology, has been implicated in various health outcomes. The correlation between strontium and other heavy metals such as lead and arsenic may reflect shared environmental sources or similar biological pathways of metabolism and excretion [25].

The lack of significant correlations for other trace elements may indicate differences in their metabolic processing, routes of exposure, or variability in biological half-lives. This finding highlights the complexity of interpreting exposure data and suggests that multiple biomarkers may be necessary to accurately assess the burden of different metals [26].

The implications of these findings may be useful for public health and environmental monitoring. By establishing the correlations between the urinary and blood levels of these trace elements, it may be possible to streamline biomonitoring efforts, allowing for more accessible and less invasive methods of assessing exposure in children. This approach could enhance our understanding of the health impacts associated with environmental exposures and inform interventions aimed at reducing these risks.

Future research should focus on longitudinal studies to monitor changes over time and explore the health effects of chronic exposure. Additionally, investigating the sources and pathways of exposure can inform targeted interventions. Understanding the role of dietary habits, environmental factors, and socioeconomic status in trace element exposure will be crucial in developing comprehensive public health strategies.

When interpreting our findings, it is important to carefully consider the potential limitations inherent in a cross-sectional pilot biomonitoring design. Our study utilized a self-recruitment strategy within schools, where eligible children were receiving education. Consequently, the sample of girls and boys evaluated may not be fully representative of the source population. We must also acknowledge that we were unable to capture the full spectrum of intrinsic and extrinsic factors that may influence trace element levels in blood and urine.

The 24 h exclusion of processed foods may overlook chronic dietary intake patterns. As processed food consumption can influence background metal exposure, particularly for blood biomarkers, this exclusion criterion could limit the generalizability of findings to populations with frequent processed food consumption.

We analyzed first-morning urine samples collected after 24 h dietary restrictions to minimize diurnal and dietary variability, a standard pediatric biomonitoring practice. While this approach provides standardized comparability with fasting blood samples, it may not reflect long-term exposure patterns.

Additionally, in this pilot biomonitoring study, we focused primarily on assessing exposure levels to toxic elements, in line with its exploratory scope. Consequently, the study did not evaluate associated health effects in the population. This limitation was necessary to prioritize methodological validation and baseline exposure characterization, which are critical for guiding future, larger-scale investigations. Future studies should integrate health outcome assessments to establish direct links between the observed exposures and potential clinical or subclinical effects.

## 5. Conclusions

Our results suggest that urinary sampling may represent a viable noninvasive alternative to blood collection for monitoring certain trace elements (e.g., lead) in pediatric populations, particularly in large-scale epidemiological studies where participant compliance is essential. However, the modest correlation strengths observed for other elements underscore the necessity of element-specific validation before urine can be universally adopted as a biomonitoring matrix. Further research should investigate the pharmacokinetic determinants and exposure dynamics underlying these associations to refine noninvasive biomonitoring strategies for assessing environmental health risks in children.

## Figures and Tables

**Table 1:** Blood and urinary levels and their correlation in analyzed school-aged children, Mexico, 2023.

	Median (IQR), ng/mL		Rho (95% CI), p
	**Blood**	**Urine**	
Aluminum	327.9 (216.2–612.7)	7.9 (4.7–13.3)	0.11 (−0.10–0.31), 0.303
Arsenic	21.4 (10.6–27.8)	13.4 (5.9–19.6)	0.23 (0.01–0.44), 0.039
Barium	1.9 (1.1–3.9)	1.8 (1.0–2.5)	0.04 (−0.16–0.24), 0.688
Cesium	2.8 (2.3–3.3)	8.0 (4.6–11.0)	0.19 (−0.01–0.39), 0.066
Cobalt	0.6 (0.1–0.7)	1.2 (0.8–1.6)	0.20 (−0.02–0.41), 0.081
Copper	1083.4 (1055.8–1127.0)	9.6 (4.3–19.0)	−0.01 (−0.23–0.21), 0.930
Iodine	53.0 (47.1–63.9)	178.7 (118.9–255.3)	0.08 (−0.12–0.29), 0.437
Lead	11.3 (4.0–23.8)	1.8 (1.5–2.0)	0.43 (0.24–0.61), <0.001
Lithium	2.1 (1.5–2.8)	41.9 (30.6–53.6)	−0.19 (−0.40–0.01), 0.063
Manganese	15.5 (12.0–18.5)	0.1 (0.1–0.6)	−0.11 (−0.33–0.11), 0.327
Molybdenum	5.0 (4.2–6.1)	93.6 (63.4–131.2)	0.10 (−0.12–0.32), 0.374
Nickel	5.6 (3.7–8.6)	7.6 (4.8–9.1)	0.01 (−0.19–0.20), 0.946
Selenium	161.0 (147.7–178.1)	63.6 (56.7–87.4)	0.20 (−0.01–0.41), 0.069
Strontium	46.2 (39.2–63.8)	157.8 (111.1–257.5)	0.22 (0.03–0.40), 0.023
Tellurium	0.3 (0.3–0.3)	0.59 (0.58–0.61)	−0.01 (−0.23–0.22), 0.979
Titanium	23.3 (15.4–30.7)	22.0 (15.4–35.7)	−0.05 (−0.27–0.17), 0.658
Zinc	5496 (5127–5784)	604.8 (427.1–822.0)	−0.08 (−0.30–0.14), 0.474

Abbreviations: IQR, interquartile range; CI, confidence interval.

## Data Availability

Data requests should be directed to the corresponding author and will be reviewed by the lead investigators and the funding council.

## Supplementary Materials

The following supporting information can be downloaded at: https://www.mdpi.com/article/10.3390/toxics13060431/s1, Supplementary Data: Optimization parameter and equipment conditions for ICP-MS analysis.

## Abbreviations

The following abbreviations are used in this manuscript:

CINVESTAV	Center for Research and Advanced Studies of the National Polytechnic Institute
EDTA	Ethylenediaminetetraacetic acid
LOD	Limit of detection
SECIHTI	Ministry of Science, Humanities, Technology, and Innovation
