# Supplementary material for: The Screening and Correlation of Trace Elements in the Blood and Urine of School-Aged Children (5–12 Years): A Pilot Biomonitoring Study
Source: Toxics. 2025 May 25;13(6):431. doi: 10.3390/toxics13060431 (PMC12197559; doi:10.3390/toxics13060431)
Supplement: Supplementary file 1 [file toxics-13-00431-s001.zip › toxics-3567081-supplementary.pdf]

Screening and Correlation of Trace Elements in Blood and Urine of School-Aged Children (5–12 Years): A Pilot Biomonitoring Study

Supplementary data:

Optimization parameter and equipment conditions for ICP-MS analysis

Optimization parameters

Parameter	Intensities
Be-9 m/z	> 4,000 cps
In-115 m/z	> 28,000 cps
U-238 m/z	> 20,000 cps
Bkgd	< 2.5 cps
**Relation**	**%**
Ce++/Ce	< 4
CeO/Ce	< 3
**RSD**	< 0.02

Equipment conditions

Parameter	Setting
**Instrument**	
Nebulizer Gas Flow	0.8 L min^{-1} Ar
Auxiliary Gas Flow	1.2 L min $^{-1}$ Ar
Plasma Gas Flow	18 L min $^{-1}$ Ar
RF power	1600
Vacuum Pressure	< 1^{-8} torr
**Timing**	
Scan mode	Standard
Sweeps/Reading	20
Readings/Replicate	1
Replicates	3
Dwell time	50 ms
Detector mode	Dual
Calibration regression type	Simple Linear
**Method**	
Sample Flush	35 s
Read Delay	15 s
Wash	45 s
